# On Serine Octamer Substitution Reactions

**DOI:** 10.1002/rcm.70182

**Published:** 2026-09-16

**Authors:** Brison A. Shira, Alana K. E. Thomas, R. Graham Cooks

**Affiliations:** ^1^ Department of Chemistry Purdue University West Lafayette Indiana USA

**Keywords:** chiral analysis, ion trap mass spectrometry, molecular clusters, serine octamer

## Abstract

**Rationale:**

Monomeric amino acids substitute into the ionized serine octamer (Ser_8_), a magic number cluster that exhibits chiral behavior. Small biomolecules, such as amino acids (AA), substitute into the octamer enantioselectively, giving Ser_7_AAH^+^ and Ser_6_AA_2_H^+^ favoring clusters of all L‐ or all D‐chirality. While the formation of Ser_8_H^+^ has been well studied, its substitution reactions need further investigation. The fact that water microdroplets readily produce Ser_8_H^+^, which undergoes enantiospecific substitution reactions perhaps gives this topic relevance to origins of life chemistry.

**Methods:**

The substitution reactions of several proteogenic AA's (Ala, Leu, isoLeu, Pro, Phe, and Val), along with prebiotically relevant isomers (norVal and isoVal), were investigated using a quadrupole ion trap mass spectrometer to gain insight into the determinants of the chiral behavior. Rather than using isotopically labeled enantiomers, as in previous work, cluster formation was assessed using samples of variable chiral composition and concentration.

**Results:**

Substitution enantioselectivity was confirmed, though in the case of some double substitutions and the substitution of nonproteogenic isomers, the homochiral preference was decreased or inverted, an observation which is attributed to the disruption of the highly ordered noncovalent contacts of the parent Ser_8_ cluster. Additionally, the data suggest that octamer formation is consecutive: Ser_8_H^+^ forms first, followed by substitution of AA to form Ser_7_AAH^+^, sometimes followed by a second substitution to form Ser_6_AA_2_H^+^.

**Conclusions:**

This investigation illustrates how chiral chemistry using MS must operate through weak but chirality‐sensitive forces while avoiding dominance of stronger but chirality‐insensitive reaction drivers; namely the propensity of a chiral organic base to acquire the charge of a proton affiliated with other chiral molecules. These results are significant because chiral molecular clusters are fundamentally interesting. Understanding the origins of their enantioselectivity contributes to a general understanding of how small molecules may recognize chirality in reaction partners and accumulate enantiomeric excess.

## 1 Introduction

Chiral chemistry accessed with MS is analytically significant, in spite of the fact that MS cannot differentiate enantiomers by mass‐to‐charge ratio (*m/z*) alone [1, 2]. Secondary variable(s) allow distinctions arising from interactions with chiral auxiliaries [3]. Examples include the competitive fragmentations in the kinetic method of chiral analysis [4], ion mobility measurements [5, 6], spectroscopic interrogation [7, 8, 9, 10], and direct ion trap‐based chirality differentiation [11]. Observing enantiodifferentiating chemical processes in the gas‐phase, though challenging, remains worthwhile because gas‐phase organic molecules may be accessed without any interference from solvent or the constraints of a crystal lattice [12, 13, 14], interferences, which dampen the effects of noncovalent interactions [15].

The reactions of the serine octamer cluster (Ser_8_) represent one instance of chiral supermolecular chemistry [16, 17] accessed with MS [18, 19, 20, 21]. Ser_8_ is known to exhibit chiral preferences in formation [22] and substitution reactions in which other small molecules, such as amino acids (AA's), cluster with Ser monomers in a manner that favors clusters of all L‐ or all D‐monomers [23]. These findings have been taken as evidence that noncovalent interactions between prebiotically relevant chiral molecules represent a potential explanation for how biomolecules accumulated asymmetry on the way, eventually, to biological homochirality [24, 25, 26, 27].

Our investigation uses the systematic variation of chirality and stoichiometry of several AA's to probe the substitution reactions that occur in Ser_8_ molecular clusters. The previously reported enantioselectivity of substitution [23] is confirmed and the data suggest that such reactions proceed sequentially: Ser_8_H^+^ first forms, then Ser_7_AAH^+^, potentially followed by Ser_6_AA_2_H^+^. Steric and stoichiometric effects were also observed, which had an unusual effect on the enantioselectivity of the substitution reactions, causing decreased chiral preference and a preference against homochirality [28].

## 2 Methods

Experiments were designed based on varying reagent concentration, which can be thought of as a fixed‐time kinetic method [29]. By increasing [AA] (the concentration of AA), a greater degree of reaction will be observed over a fixed time, thus allowing the sampling of a later point on the kinetic coordinate. Because this method assumes that [Ser] is in excess (always 8 mM) and the ratio of AA/Ser is not constant, quantitative measurements cannot be made, but by studying an array of AA's, useful information can be gathered. This approach differs from investigations, which use spectroscopic interrogation of the octamer [30, 31] and other clusters [32, 33, 34]. Though spectroscopic experiments generate data that can be matched with computationally determined structures, their scope and throughput is limited; fitting ab initio computations to an action spectrum acquired at a user facility is laborious [35, 36]. There is no doubt that chiral molecular clusters, including those beyond Ser_8_ [37, 38, 39], will continue to be an important research area [40, 41, 42] but the chemical space of interesting molecular clusters is vast: many clusters derived from Ser_8_ have been identified (Figure S1)—privileging experiments that can cover a greater number of combinations faster.

**FIGURE 1:**
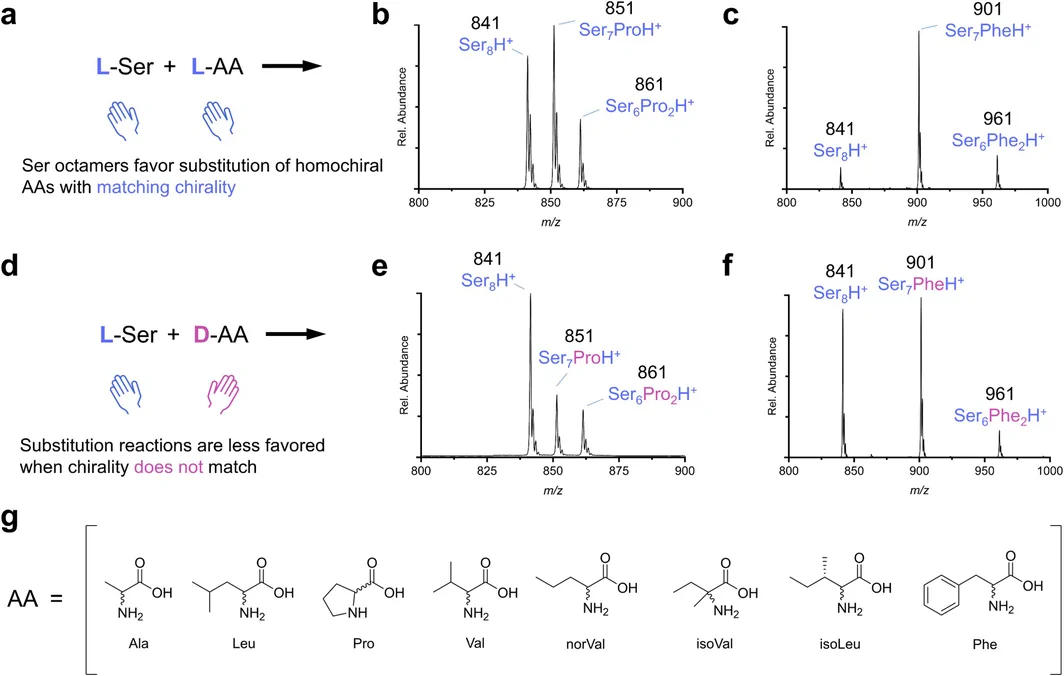
Graphical summary. The chirality of a substituting amino acid (AA) influences the preferred substitution reaction. (a) When AA matches the chirality of Ser, substitution is more likely than (d) when the chirality is mismatched. Data are from sprayed solutions of 8 mM L‐Ser with 2 mM (b and e) Pro and (c and f) Phe. (g) Structures of substituting AA's studied.

Nanoelectrospray ionization (nESI) was used to study substitution by varying the concentration of the substituting AA. Data are presented as scatter plots with *n* = 3 replicates and error bars representing two sample standard deviations. Experimentally, D‐ or L‐Ser was constituted together in LC–MS water (Fisher Scientific, Fair Lawn, NJ USA) with the AA's (Figure 1c) also of variable chirality (Millipore Sigma, St. Louis, MO USA). Replicating the results with both L‐ and D‐Ser validates the method. Ser was always 8.00 mM; AA's were used at concentrations from 1.00 to 8.00 mM; sample pH was adjusted to 1.5 using hydrochloric acid. Adjustment of pH ensured that the AA's in solution were in the +1 charge state (Figure S2). Peak assignments were confirmed with MS/MS characterization (Figures S3–18). Analyte solutions (4.0 μL aliquots) were loaded into pulled borosilicate capillary emitters with 5‐μm inner tip diameter and positioned 1.5 cm from the inlet (capillaries and P97 puller, Sutter Instrument, Novato, CA, USA). A platinum electrode was used to apply +2.0 kV spray voltage. Measurements used a Thermo LTQ XL linear quadrupole ion trap mass spectrometer with He bath gas. Automatic tuning was used to optimize the ion optics for the Ser_8_H^+^ ion, *m/z* 841. A modified full scan was used: ions were injected until the automatic gain target was met and then trapped with no supplemental activation for 1250 ms before mass analysis, improving the signal‐to‐noise by allowing metaclusters to fragment (Figure S19) [43, 44]. In the case of serine octamers, increasing the droplet flight time by changing the spray distance did not influence the substituted serine octamer formation (Figure S20). Representative mass spectra obtained for each AA are arrayed in the Supporting Information (Figures 21–29).

## 3 Results and Discussion

A range of small molecules has been demonstrated to undergo Ser_8_ substitution reactions (Table S1) [23]. Often these are observed through the protonated clusters:

(1) $${\mathrm{Ser}}_{8}H^{+}\;+\mathrm{nAA}\to {\mathrm{Ser}}_{8-n}{\mathrm{AA}}_{n}H^{+}+{\mathrm{Ser}}_{n}$$

While these reactions are well known, relatively little mechanistic information is available about them, despite several studies concerning ionized forms of Ser_8_ itself [45, 46, 47]. What is known from ion/molecule reactions [48] and studies with sonic spray ionization [49] is that proton affinity (PA, the negative of the enthalpy of protonation) can drive ligand switching reactions and that homochiral substitutions are generally more favored than substitutions involving mismatched chirality (as confirmed in Figure 1). Recent work with hydroxy acids, which have low PA, has identified the importance of α‐ and β‐hydrogen bonding contacts to serine octamer substitution reactions [28]. Molecules capable of zwitterionic salt bridge interactions exhibit the strongest clustering interactions, but even in the case of nonzwitterionic hydroxy acids, enantioselective reactivity is observed. Besides noncovalent cluster formation processes, another interesting prospect of substitution reactions concerns the possibility of using these clusters for asymmetric bond forming reactions. Past work suggests that Ser_8_ can direct the asymmetric formation of dipeptides from Pro and Leu [27]. These prior studies emphasize the need to better understand the mechanism of substitution reactions.

### 3.1 Proline Substitution

**FIGURE 2:**
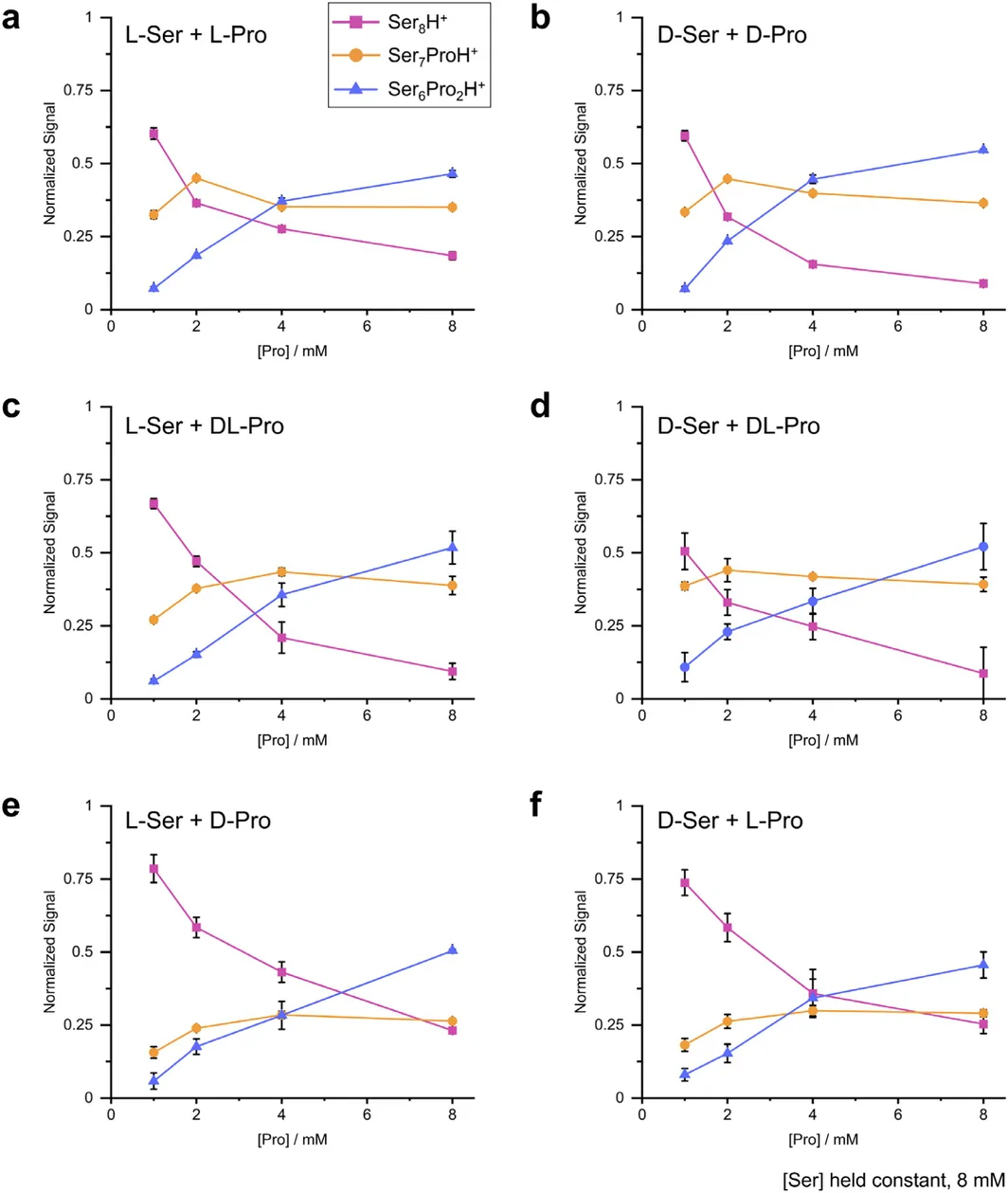
Scatter plots showing the relative abundance of Ser_8_ and Pro‐substituted octamers as [Pro] is varied. In this and all the figures that follow, abundance is given as a fraction of the total signal so the sum of all three signals equals one. (a,b) When the chirality of Ser and Pro match, the abundance of Ser_8_ is less than in the cases where the chirality does not match (e,f); this is because Ser_8_ is depleted by the favorable substitution of a matched chirality Pro to form Ser_7_ProH^+^ and Ser_6_Pro_2_H^+^.

The concentration and chirality of Pro were systematically varied allowing the relative propensity and the chirality of the substitution reactions to be observed (Figure 2). By comparing the effects of the concentration of reagents at the start of a reaction (in the bulk sprayed solution) and monitoring the effect on the distribution of products (seen as peaks in the mass spectrum), the effects of chirality and AA identity were studied.

In those cases where the chirality of Ser and Pro match (Figure 2a,b), the double substituted octamer, Ser_6_Pro_2_H^+^, is the most abundant cluster once [Pro] reached 4 mM in the spray solution. This observation confirms that substitution is enantioselective because when either racemic (DL) or mismatched Pro is used at the same concentration, Ser_6_Pro_2_H^+^ is not as abundant (Figure 2c–f). Another detail that confirms the enantioselectivity of the substitution reactions is the predominance of the Ser_8_H^+^ peak at the lowest [Pro] when the substituent chirality is mismatched (Figure 2e,f), which signals that substitution is not as favorable as in Figure 2a,b. Further evidence for the enantioselectivity of Pro substitution into the octamer is taken from the intermediate effect observed in the cases with racemic Pro (Figure 2c–d). The data also suggest consecutive substitution, in which the first substitution product, Ser_7_ProH^+^, is depleted as the second substitution product, Ser_6_Pro_2_H^+^, is generated. This is apparent in the arrayed scatter plots (Figure 2), which show first the emergence of Ser_7_ProH^+^ then, as [Pro] increases, Ser_6_Pro_2_H^+^, with the intensity of Ser_8_H^+^ decreasing throughout.

An interesting feature is that at the highest [Pro] (8 mM), the octamer products have similar distributions, regardless of their chirality. Under these conditions it is likely that competitive ionization effects dominate. The essence of this argument is similar in its logic to ion suppression effects [50, 51]. While the most kinetically advantaged homochiral substitution reactions are observed when [Pro] is lower, when [Pro] is higher, these are overcome by the favorable thermodynamics (viz. Le Chatelier's principle) that vary less strongly with chirality. With an abundance of Pro monomers available, the charge of Ser_8_H^+^ is susceptible to reaction with the more basic Pro, which has a secondary amine with a high PA of 921 kJ/mol [52]. This observation highlights the fact that in order to accomplish asymmetric chemistry in the gas‐phase, the two competing forces of chirally sensitive kinetics and chirally insensitive thermochemistry need to be balanced. Charge–charge interactions, quantified through thermochemical quantities such as PA, are usually much stronger but less selective than hydrogen bonding and steric effects which have more susceptibility to chirality.

### 3.2 Leucine and Isoleucine Substitution

**FIGURE 3:**
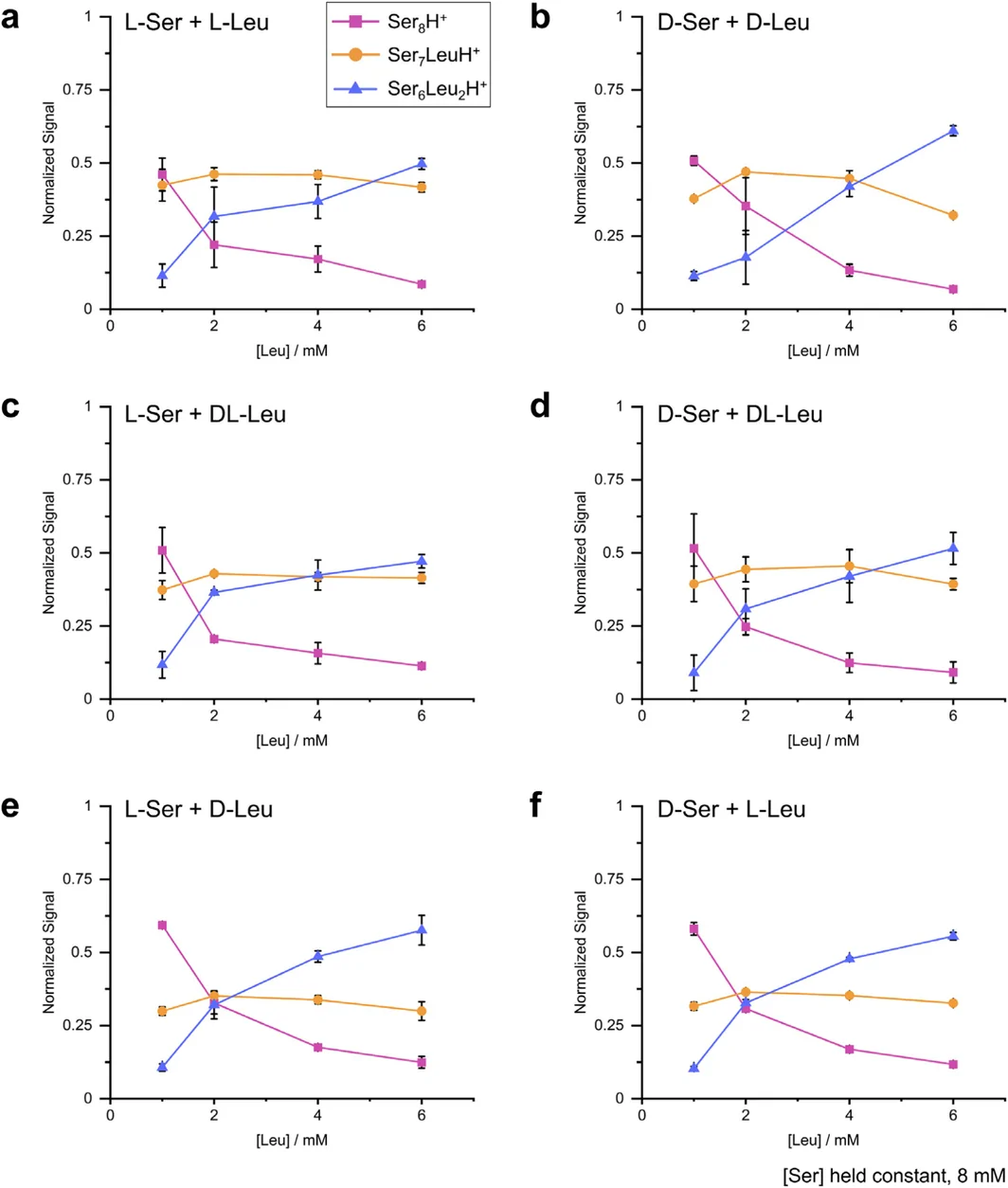
Scatter plots showing the relative abundance of Ser_8_ and Leu‐substituted octamers with varying [Leu]. (a,b) When Ser and Leu are chirality matched, Ser_7_LeuH^+^ is more abundant at lower [Leu]. In these plots the abundance of Ser_6_Leu_2_H^+^ is less than that observed when chirality does not match (e,f), illustrating a rare instance when the chiral preference inverts to favor mismatched, not matched chirality.

The substitution of Leu shares some features with that of Pro (Figure 3). Here, as with Pro, when the chirality of Leu does not match Ser and [Leu] is low (Figure 3e,f), Ser_8_H^+^ has higher relative abundance than when the chirality is matched (Figure 3a,b). Additionally, when [Leu] is highest (6 mM), the distribution of serine octamers generated is similar, regardless of the sample's chiral composition, and the double substitution to give Ser_6_Leu_2_H^+^ predominates. The same effect, though to a lesser extent, is observed for isoLeu (Figure S30). The main difference between the serine octamers obtained from Leu substitution and isoLeu substitution is that the Leu‐substituted octamers are more abundant than the parent Ser_8_H^+^ cluster. The abundance of Ser_8_H^+^ is greater when measured against the isoLeu substitution products, implying that branching on the β‐carbon may disrupt the accommodation of this AA into the cluster.

The difference between the Leu and Pro substitutions centers on the second substitution (compare Figures 2e,f and 3e,f). At low [Leu], substitution is more prominent in the homochiral cases but at higher [Leu], the usual enantioselective behavior of serine octamer substitution does not hold. It is possible that the steric profiles of Leu and isoLeu, which have bulky sidechains, disrupt the octamer's tightly packed structure and lessen or even invert the chiral preference of the substitution reaction, a phenomenon which has been observed in other molecular clusters [38, 39]. On the other hand, the structure of Pro is more rigid, locking in the polar moieties about the chiral center. Indeed, in biology Pro‐containing moieties are associated with tight turn structures [53], which may be similar to the close contacts exhibited within serine octamers. While Leu has a bulky sidechain that may disrupt the cluster's structure, Pro better maintains the contacts between the protonated amine and deprotonated carboxylic acid.

### 3.3 Alanine Substitution

In the case of Ala (Figure S31), only Ser_8_H^+^ and Ser_7_AlaH^+^ were observed. It appears that Ser_7_AlaH^+^ is less favorable than Ser_8_H^+^, as even in the case of 8.00 mM, where the concentrations of the Ser and Ala are equal, Ser_7_AlaH^+^ is not abundant, making any measured differences due to chirality small. This likely follows from Ala's reduced ability to participate in intermolecular hydrogen bonding/ion‐dipole interactions: It does not have a polar sidechain and has a slightly lower PA than Ser, 902 versus 907 kJ/mol [52]. Nevertheless, these data also confirm the enantioselectivity as previously discussed for Leu and Pro; more Ser_7_AlaH^+^ was observed when the chirality of Ala and Ser matched. Unlike the other AA's, the abundance of the substituted octamer did not always increase with [Ala]. For the chirally mismatched substitution, Ser_7_AlaH^+^ is most abundant at 4.00 mM and less so at 8.00 mM. It is possible that alternative avenues consume the Ala (such as self‐aggregation by formation of proton‐bound clusters of Ala [54, 55]) preventing it from appearing as a substitution product.

### 3.4 Valine, Isovaline, and Norvaline Substitution

**FIGURE 4:**
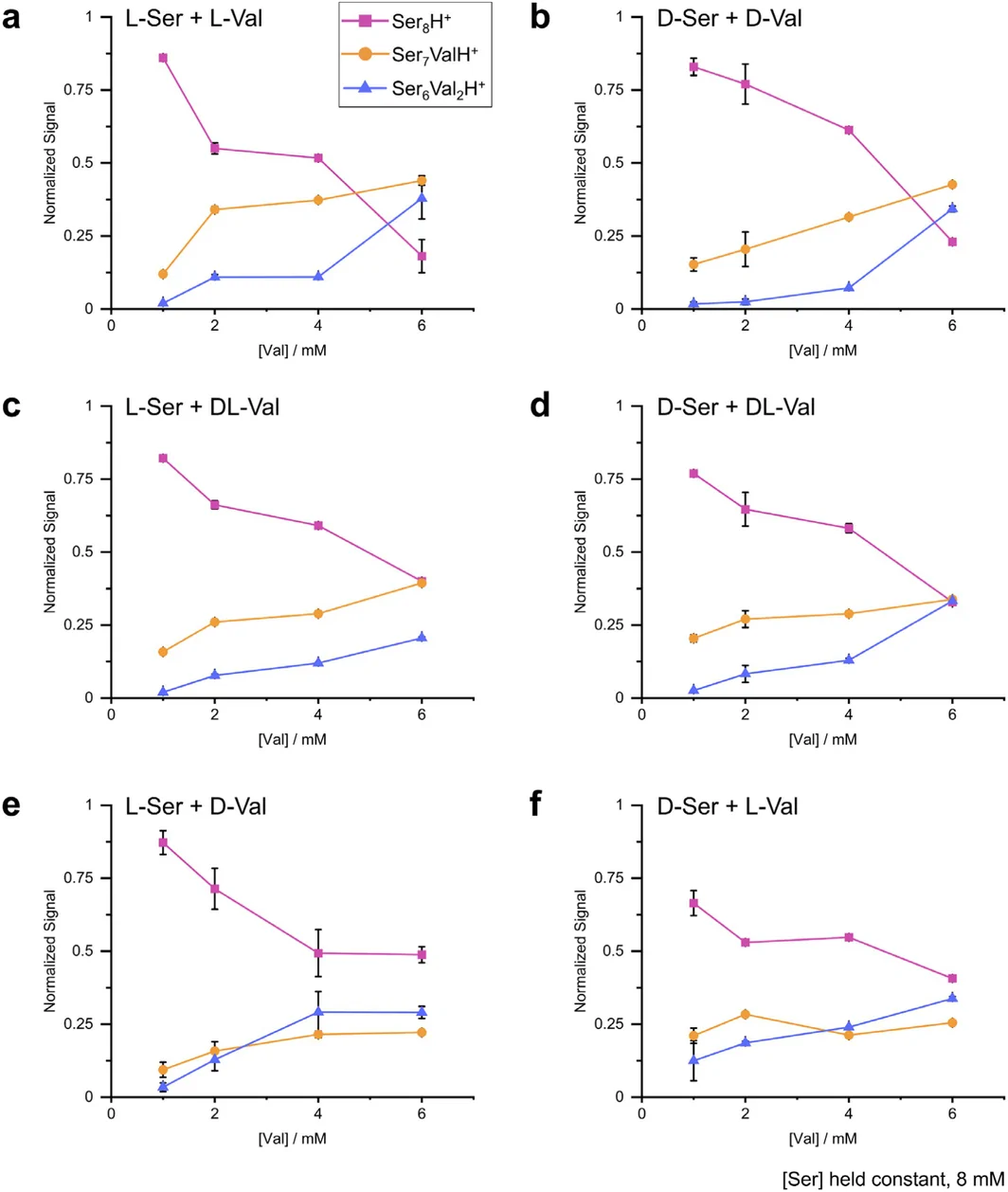
Scatter plots showing the relative abundance of Ser_8_ and Val‐substituted octamers as [Val] is varied. When Ser and Val chirality match (a,b), the single substitution is favored and the double is present only at relatively low levels. Ser_7_LeuH^+^ is more abundant at lower [Leu]. Again, as in the case of Leu (Figures 3 and S30), double substitution is more abundant than single substitution when mismatched chirality is used with > 4 mM [Val] (e,f).

The steric bulk of Leu and isoLeu's alkyl R‐groups may explain the increased abundance of the double substitution when chirality is not matched (Figures 3e,f and S30e,f). Val and its isomers, isoVal and norVal, provide further evidence for this explanation. While the single substitution to form Ser_7_ValH^+^ exhibits the expected enantioselective behavior (Figure 4a,b), the double substitution exhibits an inverted preference, making it a more abundant species at 4 and 6 mM [Val] in the case where Ser and Val have opposite chirality (Figure 4e,f). The same trend is reproduced with the isomer norVal, which has a straight chain, not a branched alkyl substituent (Figure S32).

**FIGURE 5:**
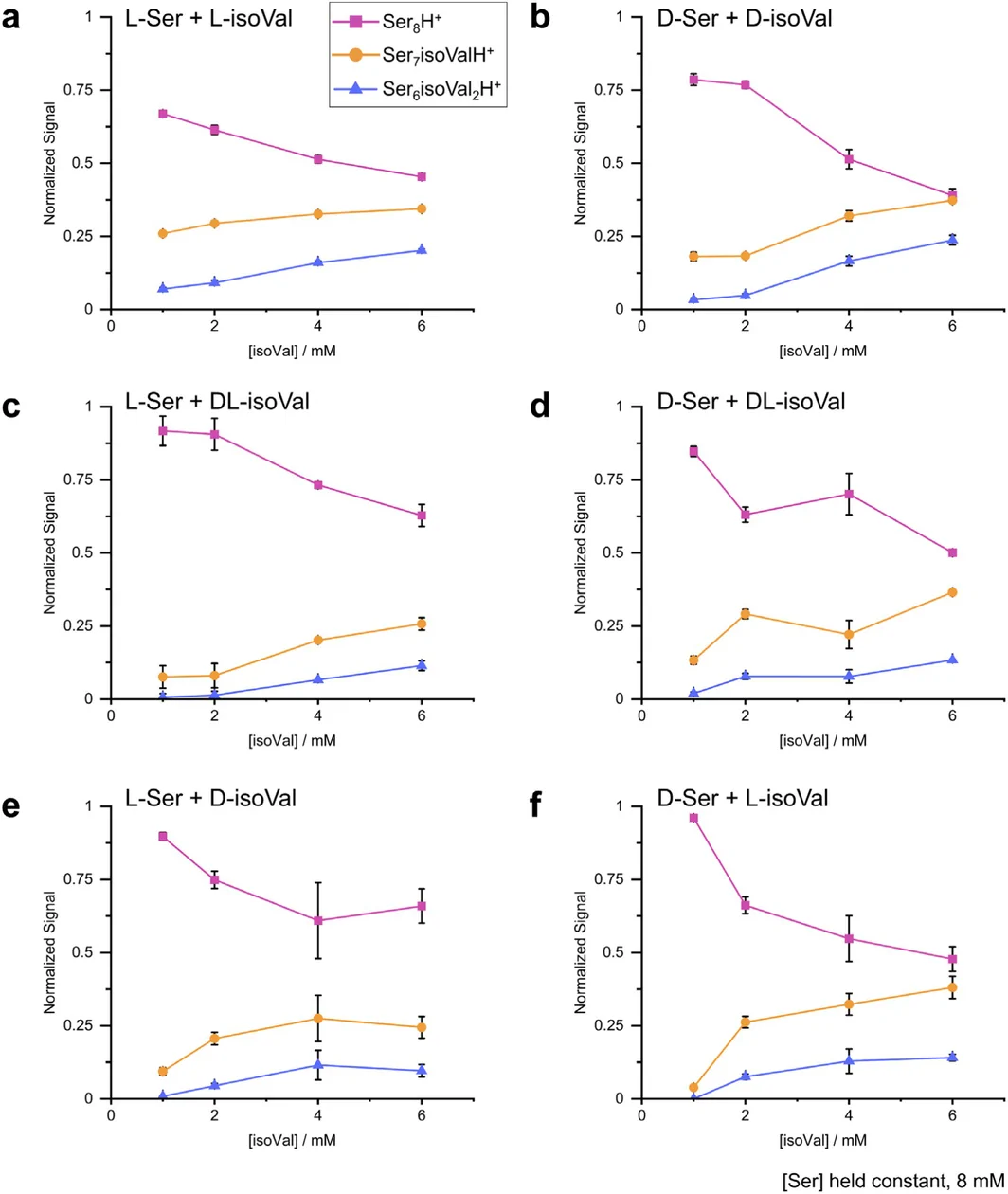
Scatter plots showing the relative abundance of Ser_8_ and isoVal‐substituted octamers as [isoVal] is varied. Strong enantiomeric discrimination is not observed in this case, which is attributed to the addition of a methyl group on the α‐carbon, which impedes the chiral recognition characteristic of the unsubstituted octamer.

For isoVal (Figure 5), which has a methyl group added to its α‐carbon where a hydrogen would be in a proteogenic AA, substitution does not appear to be consecutive because both substitutions increase in a monotonic way, nor does substitution appear to be strongly enantioselective. Varying the chirality of Ser and isoVal does not significantly influence the observed abundance of the substituted octamers. This result shows that the method used in this study can distinguish enantioselective and nonenantioselective substitutions. Studying nonproteogenic AA's and the differences of their behavior in abiotic processes, such as molecular cluster formation, may help elucidate the chemical evolution that selected the proteogenic AA's. Findings from simulated prebiotic chemistry and meteorites have resulted in a consensus view that life must have evolved in a chemical space that contained a variety of organic monomers, including the proteogenic amino acids, but also related hydroxy acids, and other “noncanonical” chemicals [56, 57]. Val, norVal, isoVal, and isoLeu are especially germane in this context because these have been identified in meteorite samples in a chirally enriched form [58, 59, 60, 61]. It is interesting then that isoVal, which is not a proteogenic AA, is unable to participate in a proposed enantio‐amplification scheme [1].

### 3.5 Phenylalanine Substitution

**FIGURE 6:**
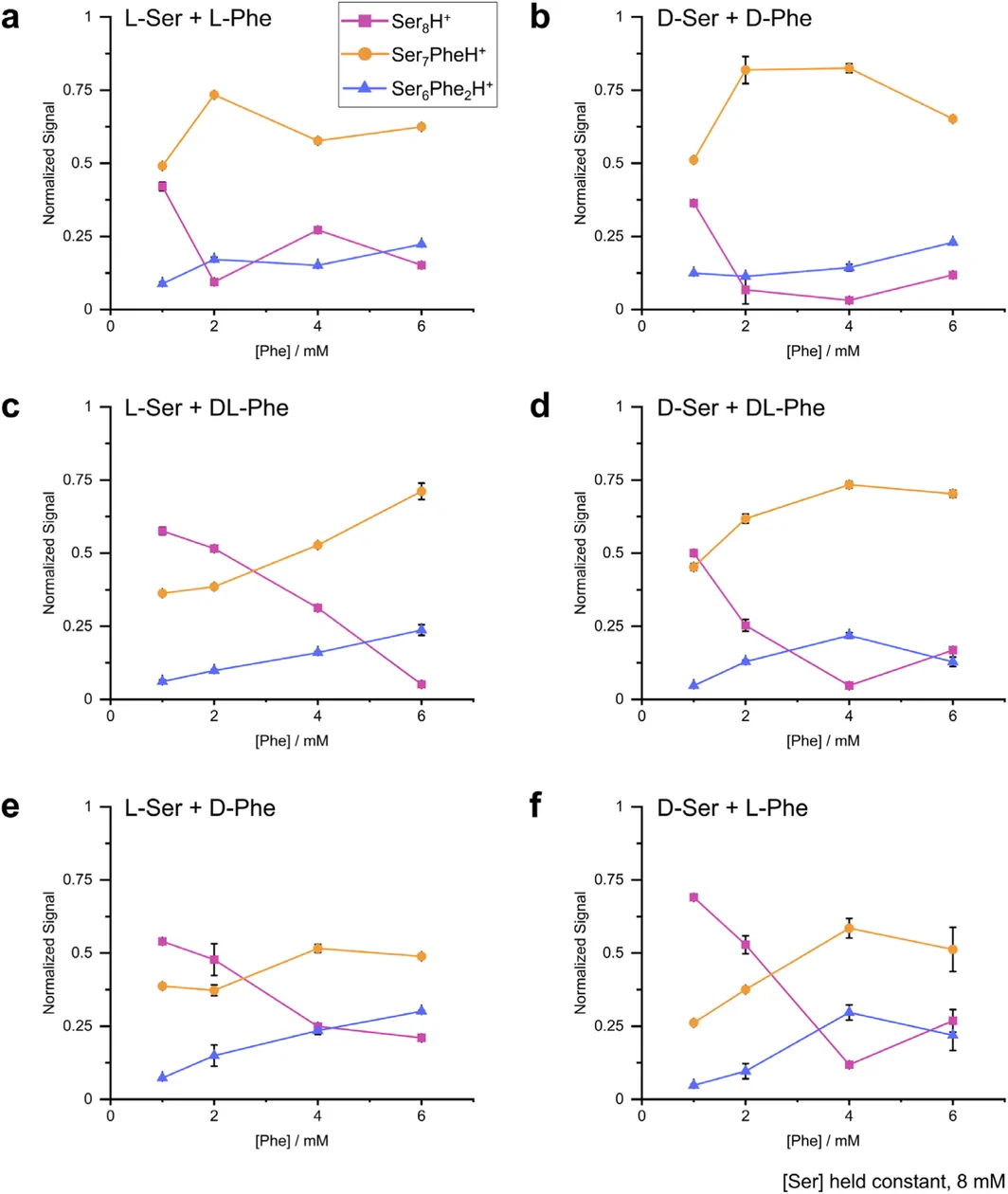
Scatter plots showing the relative abundance of Ser_8_ and Phe‐substituted octamers as [Phe] is varied. When Ser and Phe chirality match (a,b), Ser_7_PheH^+^ is the most abundant species. The double substitution however, appears at similar abundance in all cases, irrespective of the chirality of the Ser and Phe.

Of the AA's studied, Phe exhibits the most abundant single substitution at low [AA]. In the cases in which Ser and Phe have matching chirality (Figure 6a,b), Ser_7_PheH^+^ is the most abundant octamer. Here, as with Val and Leu, the double substitution is most abundant when the chirality of Ser and Phe are mismatched (Figure 6e,f). The favorable and enantiospecific formation of Ser_7_PheH^+^ represents the goldilocks condition where thermochemistry (the enthalpic drive of an organic base to acquire a charged proton) is relatively less important than the chiral noncovalent contacts, which give Ser_8_ its enantiospecificity. The PA of Phe is 906 kJ/mol, which is close to that of Ser, 907 kJ/mol [52]. Because the exchange of a Ser monomer for a Phe in the cluster has only small thermochemical consequence, the relative importance of the chiral noncovalent contacts increases, a factor compounded by the steric bulk of Phe.

## 4 Conclusions

These data indicate (i) the basic fact of enantiomeric control of substitution, at least in cases where excessive concentrations do not overwhelm the delicate gas‐phase intermolecular interactions through which the chiral effect is observable; (ii) homochiral serine octamer substitutions are consecutive; (iii) charge stabilization may influence the observed abundance of a given substitution; and (iv) the substitution of AA's with sterically bulky groups (e.g., double substitution of Leu, Val, and norVal) can in some cases decrease or even invert the homochiral preference of octamer substitutions, while the addition of a nonhydrogen function on the α‐carbon can dampen enantioselection. A sequence of reactivity was measured in which the AA's most likely to engage in substitution are: Phe > Pro > Leu > Val > isoLeu > isoVal > norVal > Ala, with Phe, Pro, and Val exhibiting the strongest enantiodifferentiation.

Microdroplets, the medium from which serine octamers were generated, exhibit many interesting and useful properties. Indeed, droplets and related chemical environments have long been hypothesized to play a role in life's origin and chiral chemistry [62, 63, 64]. The octamer likely forms during the evaporation of the microdroplets that deliver analyte from the nESI emitter to the vacuum chamber [45, 65]. Because microdroplets are a unique chemical environment and potentially are “abiotic reactors” [66] where acid/base chemistry is different than in bulk, examining the effect of thermochemical drivers like PA on chiral cluster formation is reasonable. Therefore, spray ionization MS is an advantaged experiment for the examination of chiral clusters because it offers ready access to the products of (chiral) droplet chemistry [46, 67, 68].

MS‐based chiral analysis matters because MS provides an avenue for simple and rapid exploration [69] of chirality and how chiral effects manifest biologically [70, 71, 72]. Though the understanding of the role of charge in the reactions of the octamer is not complete, it is interesting to consider the suggestion that neutral Ser_8_, not Ser_8_H^+^, is the underlying magic cluster [73] although the aqueous microdroplets have strongly acidic (or in the opposite polarity, basic) interfaces and ionic forms might have been directly involved in the prebiotic process of chiral selection. The structure of Ser_8_H^+^ does not feature higher order symmetry as protonation disrupts the hydrogen bonding/ion‐dipole interactions [36]. However, the dichloride octamer, which has more polarizable chloride ions [74], features D_4_ symmetry [35], raising the question of whether neutral clusters have such symmetry and exhibit even better enantioselectivity. In either case, the serine octamer cluster provides an avenue through which enantioselected material can be transported differently than nonselected material, a potential enrichment scheme [1, 73]. The importance of this phenomenon motivated this investigation's illustration of the fact that gas‐phase chiral chemistry involves two competing chemical forces weak but chirality sensitive sterics and hydrogen bonding and on the other hand strong but chirality insensitive drivers such as PA.

## Author Contributions

**Brison A. Shira:** investigation, methodology, writing – original draft, conceptualization, validation, visualization, writing – review and editing, formal analysis. **Alana K. E. Thomas:** formal analysis, visualization, validation, methodology, investigation. **R. Graham Cooks:** methodology, supervision, project administration, writing – review and editing, writing – original draft, conceptualization, investigation, funding acquisition, validation, visualization, formal analysis, resources.

## Funding

This work was supported by Multidisciplinary University Research Initiative (FA9550‐21‐1‐0170 and 62741613‐204669) and Agilent Technologies.

## Supporting information


**Table S1:** Serine octamers identified in the literature.
**Figure S2:** pH optimization of nESI for substitution in Ser8 by Ala.
**Figure S3:** MS/MS confirming assignment of *m/z* 841 as Ser8H^+^.
**Figure S4:** MS/MS confirming assignment of *m/z* 825 as Ser7AlaH^+^.
**Figure S5:** MS/MS confirming assignment of *m/z* 851 as Ser7ProH^+^.
**Figure S6:** MS/MS confirming assignment of *m/z* 861 as Ser6Pro2H^+^.
**Figure S7:** MS/MS confirming assignment of *m/z* 867 as Ser7LeuH^+^.
**Figure S8:** MS/MS confirming assignment of *m/z* 893 as Ser6Leu2H^+^.
**Figure S9:** MS/MS confirming assignment of *m/z* 853 as Ser7norValH^+^.
**Figure S10:** MS/MS confirming assignment of *m/z* 865 as Ser6norVal2H^+^.
**Figure S11:** MS/MS confirming assignment of *m/z* 853 as Ser7isoValH^+^.
**Figure S12:** MS/MS confirming assignment of *m/z* 865 as Ser6isoVal2H^+^.
**Figure S13:** MS/MS confirming assignment of *m/z* 867 as Ser7isoLeuH^+^.
**Figure S14:** MS/MS confirming assignment of *m/z* 893 as Ser6isoLeu2H^+^.
**Figure S15:** MS/MS confirming assignment of *m/z* 901 as Ser7PheH^+^.
**Figure S16:** MS/MS confirming assignment of *m/z* 961 as Ser6Phe2H^+^.
**Figure S17:** MS/MS confirming assignment of *m/z* 853 as Ser7ValH^+^.
**Figure S18:** MS/MS confirming assignment of *m/z* 865 as Ser6Val2H^+^.
**Figure S19:** Optimization of trapping time not using collision‐induced dissociation.
**Figure S20:** Distance effect in formation of Ser7AlaH^+^.
**Figure S21:** Array of representative mass spectra showing Pro substitution.
**Figure S22:** Array of representative mass spectra showing Leu substitution.
**Figure S23:** Array of representative mass spectra showing isoLeu substitution.
**Figure S24:** Array of representative mass spectra showing Ala substitution.
**Figure S25:** Array of representative mass spectra showing norVal substitution.
**Figure S26:** Array of representative mass spectra showing isoVal substitution.
**Figure S27:** Array of representative mass spectra showing Val substitution.
**Figure S28:** Array of representative mass spectra showing Phe substitution.
**Figure S29:** MS/MS of Phe‐related cluster *m*/*z* 908.5.
**Figure S30:** Scatter plot of isoLeu substitution.
**Figure S31:** Scatter plot of Ala substitution.
**Figure S32:** Scatter plot of norVal substitution.

## Data Availability

The data that support the findings of this study are available from the corresponding author upon reasonable request.
