# Supplementary material for: On Serine Octamer Substitution Reactions
Source: Rapid Commun Mass Spectrom. 2026 Sep 16;40(23):e70182. doi: 10.1002/rcm.70182 (PMC13583542; doi:10.1002/rcm.70182)
Supplement: Supplementary file 1 [file RCM-40-e70182-s001.docx]

**Supporting Information**

**On Serine Octamer Substitution Reactions**

Brison A. Shira, Alana K. E. Thomas, and R. Graham Cooks*

Department of Chemistry, Purdue University, 560 Oval Drive, West Lafayette, IN 47907

*Email: [cooks@purdue.edu](mailto:cooks@purdue.edu)

**Table of Contents**

[Table S1: Serine octamers identified in the literature	1](#_Toc236832747)

[Figure S2: pH optimization of nESI for substitution in Ser_8_ by Ala	2](#_Toc236832748)

[Figure S3: MS/MS confirming assignment of *m/z* 841 as Ser_8_H^+^	3](#_Toc236832749)

[Figure S4: MS/MS confirming assignment of *m/z* 825 as Ser_7_AlaH^+^	4](#_Toc236832750)

[Figure S5: MS/MS confirming assignment of *m/z* 851 as Ser_7_ProH^+^	5](#_Toc236832751)

[Figure S6: MS/MS confirming assignment of *m/z* 861 as Ser_6_Pro_2_H^+^	6](#_Toc236832752)

[Figure S7: MS/MS confirming assignment of *m/z* 867 as Ser_7_LeuH^+^	7](#_Toc236832753)

[Figure S8: MS/MS confirming assignment of *m/z* 893 as Ser_6_Leu_2_H^+^	8](#_Toc236832754)

[Figure S9: MS/MS confirming assignment of *m/z* 853 as Ser_7_norValH^+^	9](#_Toc236832755)

[Figure S10: MS/MS confirming assignment of *m/z* 865 as Ser_6_norVal_2_H^+^	10](#_Toc236832756)

[Figure S11: MS/MS confirming assignment of *m/z* 853 as Ser_7_isoValH^+^	11](#_Toc236832757)

[Figure S12: MS/MS confirming assignment of *m/z* 865 as Ser_6_isoVal_2_H^+^	12](#_Toc236832758)

[Figure S13: MS/MS confirming assignment of *m/z* 867 as Ser_7_isoLeuH^+^	13](#_Toc236832759)

[Figure S14: MS/MS confirming assignment of *m/z* 893 as Ser_6_isoLeu_2_H^+^	14](#_Toc236832760)

[Figure S15: MS/MS confirming assignment of *m/z* 901 as Ser_7_PheH^+^	15](#_Toc236832761)

[Figure S16: MS/MS confirming assignment of *m/z* 961 as Ser_6_Phe_2_H^+^	16](#_Toc236832762)

[Figure S17: MS/MS confirming assignment of *m/z* 853 as Ser_7_ValH^+^	17](#_Toc236832763)

[Figure S18: MS/MS confirming assignment of *m/z* 865 as Ser_6_Val_2_H^+^	18](#_Toc236832764)

[Figure S19: Optimization of trapping time not using collision-induced dissociation	19](#_Toc236832765)

[Figure S20: Distance effect in formation of Ser_7_AlaH^+^	20](#_Toc236832766)

[Figure S21: Array of representative mass spectra showing Pro substitution	21](#_Toc236832767)

[Figure S22: Array of representative mass spectra showing Leu substitution	22](#_Toc236832768)

[Figure S23: Array of representative mass spectra showing isoLeu substitution	23](#_Toc236832769)

[Figure S24: Array of representative mass spectra showing Ala substitution	24](#_Toc236832770)

[Figure S25: Array of representative mass spectra showing norVal substitution	25](#_Toc236832771)

[Figure S26: Array of representative mass spectra showing isoVal substitution	26](#_Toc236832772)

[Figure S27: Array of representative mass spectra showing Val substitution	27](#_Toc236832773)

[Figure S28: Array of representative mass spectra showing Phe substitution	28](#_Toc236832774)

[Figure S29: MS/MS of Phe-related cluster *m/z* 908.5	29](#_Toc236832775)

[Figure S30: Scatter plot of isoLeu substitution	30](#_Toc236832776)

[Figure S31: Scatter plot of Ala substitution	31](#_Toc236832777)

[Figure S32: Scatter plot of norVal substitution	32](#_Toc236832778)

[References	33](#_Toc236832779)

Table S1: Serine octamers identified in the literature

| **Year** | **Description** | **Number of octamers identified** | **Reference** (see p. S33) |
| --- | --- | --- | --- |
| 2001 | [Ser_8_ + H]^+^, [Ser_8_ + Na]^+^, [Ser_8_ + K]^+^ | 3 | 1 |
| 2002 | [Ser_16_ + 2H]^+^, [Ser_24_ + 3H]^+^  Higher clusters | 2 | 2 |
| 2003 | Octamers substituted with other amino acids: Gly, Ala, Leu, Thr, Cys, Phe, Tyr, Trp, Asp, His; all exhibit at least mono and di-substitution when chirally matched and mis-matched; these can also form with Na^+^ or K^+^ | 56 | 3  (q.v. Table 3) |
| 2004 | Chemical modifications to serine identified an alternative magic number cluster, [4 O-Acetyl-L-Ser + H]^+^ | 0 | 4 |
| 2005 | [Ser_8_ + 2Cl]^─^ and [Ser_8_ + 2Br]^─^ | 2 | 5 |
| 2021 | Similar to Ref 2, higher clusters identified, though not necessarily multiples of 8 | 0 | 6 |
| 2025 | Octamers substituted with Pro (L and D), 2 Pro (LL, DL, DD), and Pro + Leu (LL, LD, DL, DD) | 9 | 7 |
| 2026 | Octamers substituted with tartaric (L, D, meso), malic (L and D), glyceric (L and D), and succinic acid (achiral) | 8 | 8 |
| **Total*:** | | **80** | |

*Not tabulated are the octamers identified herein.

Figure S2: pH optimization of nESI for substitution in Ser_8_ by Ala

The pH of the nESI spray solution was varied to optimize the conditions used in this study. Reducing the pH to 1.5 with HCl gave the highest signal to noise for Ser_8_H^+^ and Ser_7_AlaH^+^ at *m/z* 841 and 825, respectively. The absolute intensity in the pH 1.5 spectrum is *ca.* 200 counts, in pH 4.5 it is 100 counts, and in pH 6.0 it is 200 counts.

N.B.: At pH 9 and 12, achieved with the addition of NaOH, S/N >3 was not achieved for ions of *m/z* 841 or 863. The high pH makes the observation of a protonated species unfavorable (this is sometimes called “wrong-way-round.”^9, 10^

Figure S3: MS/MS confirming assignment of *m/z* 841 as Ser_8_H^+^

This spectrum was obtained from a solution of 8.00 mM L-Ser at pH 1.5.

Figure S4: MS/MS confirming assignment of *m/z* 825 as Ser_7_AlaH^+^

This spectrum was obtained from a solution of 8.00 mM D-Ser and 4.00 mM D-Ala at pH 1.5.

Figure S5: MS/MS confirming assignment of *m/z* 851 as Ser_7_ProH^+^

This spectrum was obtained from a solution of 8.00 mM D-Ser and 4.00 mM D-Pro at pH 1.5.

Figure S6: MS/MS confirming assignment of *m/z* 861 as Ser_6_Pro_2_H^+^

This spectrum was obtained from a solution of 8.00 mM D-Ser and 4.00 mM D-Pro at pH 1.5.

Figure S7: MS/MS confirming assignment of *m/z* 867 as Ser_7_LeuH^+^

This spectrum was obtained from a solution of 8.00 mM L-Ser and 4.00 mM L-Leu at pH 1.5. Note the presence of diffuse peaks associated with the ready fragmentation of the leucine complex under near resonant conditions. This feature is not observed for the other complexes.

Figure S8: MS/MS confirming assignment of *m/z* 893 as Ser_6_Leu_2_H^+^

This spectrum was obtained from a solution of 8.00 mM L-Ser and 4.00 mM L-Leu at pH 1.5. Note, again, the presence of diffuse peaks associated with the ready fragmentation of the leucine complex under near resonant conditions.

Figure S9: MS/MS confirming assignment of *m/z* 853 as Ser_7_norValH^+^

This spectrum was obtained from a solution of 8.00 mM L-Ser and 6.00 mM L-norVal at pH 1.5.

Figure S10: MS/MS confirming assignment of *m/z* 865 as Ser_6_norVal_2_H^+^

This spectrum was obtained from a solution of 8.00 mM L-Ser and 6.00 mM L-norVal at pH 1.5. The fragment *m/z* 760, the loss of one Ser monomer, is quite abundant, suggesting that the incorporation of the two norVal monomers disrupts the structure of the octamer in such a way that the loss of two neutral serine monomers is not the preferred pathway, as in the all-serine octamer (see **Fig S3**).^8^ The peak *m/z* 866 represents off-target isolation and is not thought to compromise interpretation of the observed fragment ions.

Figure S11: MS/MS confirming assignment of *m/z* 853 as Ser_7_isoValH^+^

This spectrum was obtained from a solution of 8.00 mM D-Ser and 6.00 mM D-isoVal at pH 1.5.

Figure S12: MS/MS confirming assignment of *m/z* 865 as Ser_6_isoVal_2_H^+^

This spectrum was obtained from a solution of 8.00 mM D-Ser and 6.00 mM D-isoVal at pH 1.5. The peak *m/z* 867 represents off-target isolation and is not thought to compromise interpretation of the observed fragment ions.

Figure S13: MS/MS confirming assignment of *m/z* 867 as Ser_7_isoLeuH^+^

This spectrum was obtained from a solution of 8.00 mM L-Ser and 6.00 mM L-isoVal at pH 1.5.

Figure S14: MS/MS confirming assignment of *m/z* 893 as Ser_6_isoLeu_2_H^+^

This spectrum was obtained from a solution of 8.00 mM L-Ser and 6.00 mM L-isoVal at pH 1.5.

Figure S15: MS/MS confirming assignment of *m/z* 901 as Ser_7_PheH^+^

This spectrum was obtained from a solution of 8.00 mM L-Ser and 6.00 mM L-Phe at pH 1.5. The peak *m/z* 903 represents off-target isolation but is not thought to compromise interpretation of the observed fragment ions.

Figure S16: MS/MS confirming assignment of *m/z* 961 as Ser_6_Phe_2_H^+^

This spectrum was obtained from a solution of 8.00 mM L-Ser and 6.00 mM L-Phe at pH 1.5.

Figure S17: MS/MS confirming assignment of *m/z* 853 as Ser_7_ValH^+^

This spectrum was obtained from a solution of 8.00 mM D-Ser and 6.00 mM D-Val at pH 1.5. The peak *m/z* 855 represents off-target isolation but is not thought to compromise interpretation of the observed fragment ions.

Figure S18: MS/MS confirming assignment of *m/z* 865 as Ser_6_Val_2_H^+^

This spectrum was obtained from a solution of 8.00 mM D-Ser and 6.00 mM D-Val at pH 1.5. The peak *m/z* 867 represents off-target isolation but is not thought to compromise interpretation of the observed fragment ions.

Figure S19: Optimization of trapping time not using collision-induced dissociation

The highest signal-to-noise ratio, S/N, was achieved at trapping times around 1000 ms using the test case of D-Ser (pH 1.5, 8.00 mM). S/N was computed as follows: noise was the standard deviation of all intensity in the mass range from *m/z* 800 - 835 and signal was the maximum intensity of the peak at *m/z* 841.

Figure S20: Distance effect in formation of Ser_7_AlaH^+^

To ascertain whether a reproducible distance effect could be obtained, three replicate trials at three distances (0.3 cm, 1.0 cm and 2.0 cm) were attempted using 1.00 mM L-Ala and 8.00 mM L-Ser. At 2 cm, very low signal was obtained. Because this method did not give reliable differentiation between distances, an initial rate method was adopted as described in the main text. The distance used for the main text data, 1.5 cm, gave the best reproducibility, enabling measurements with ca. 10% relative error.

Figure S21: Array of representative mass spectra showing Pro substitution

Substitution of proline gives the octamers Ser_7_ProH^+^ and Ser_6_Pro_2_H^+^ at *m/z* 851 and 861. Spectra are arranged from (*bottom*) lowest to (*top*) highest [Pro]; horizontally by chiral composition, with (*far left*) L-Ser/L-Pro and (*far right*) D-Ser/D-Pro. For [Pro] of 1.00 and 2.00 mM with matching chirality, the monosubstitution at *m/z* 851 dominates, but it becomes less intense, at higher concentrations indicating consecutive kinetics. Substitutions are most favored when the chirality of the substituting AA matches that of serine.

Figure S22: Array of representative mass spectra showing Leu substitution

Substitution of leucine gives the octamers Ser_7_LeuH^+^ and Ser_6_Leu_2_H^+^ at *m/z* 867 and 893, respectively. Spectra are arranged from (*bottom*) lowest to (*top*) highest [Leu]_soln_; horizontally, they are arranged by chiral composition, with (*far left*) L-Ser/L-Leu and (*far right*) D-Ser/D-Leu. Note that the highest concentration used is 6.00 rather than 8.00 mM as for the other amino acids. For [Leu]_soln_ 1.00 mM, homochiral monosubstitution (*m/z* 867) is most favored, but as disubstitution increases, the preference for homochirality decreases.

Figure S23: Array of representative mass spectra showing isoLeu substitution

Substitution of isoLeu gives the octamers Ser_7_isoLeuH^+^ and Ser_6_isoLeu_2_H^+^ at *m/z* 867 and 893, respectively. Spectra are arranged from (*bottom*) lowest to (*top*) highest [isoLeu]; horizontally, they are arranged by chiral composition, with (*far left*) L-Ser/L-isoLeu and (*far right*) D-Ser/D-isoLeu.

Figure S24: Array of representative mass spectra showing Ala substitution

Alanine gave the monosubstituted octamer, Ser_7_AlaH^+^, *m/z* 825, as the only substitution product. Columns are arranged from (bottom) lowest to (top) highest [Ala]; horizontally by chiral composition, with (far left) L-Ser/L-Ala and (far right) D-Ser/D-Ala. The yield of Ser_7_AlaH^+^ increases with [Ala] when chirality matches.

Figure S25: Array of representative mass spectra showing norVal substitution

NorVal gave the substituted octamers, Ser_7_norValH^+^, *m/z* 853, and Ser_6_norVal_2_H^+^, *m/z* 865. Columns are arranged from (bottom) lowest to (top) highest [norVal]; horizontally by chiral composition, with (far left) L-Ser/L-norVal and (far right) D-Ser/D-norVal.

Figure S26: Array of representative mass spectra showing isoVal substitution

As with the other isomers of Val, isoVal gave the substituted octamers, Ser_7_isoValH^+^, *m/z* 853, and Ser_6_isoVal_2_H^+^, *m/z* 865. Columns are arranged from (bottom) lowest to (top) highest [isoVal]; horizontally by chiral composition, with (far left) L-Ser/L-isoVal and (far right) D-Ser/D-isoVal.

Figure S27: Array of representative mass spectra showing Val substitution

Val gave the substituted octamers, Ser_7_ValH^+^, *m/z* 853, and Ser_6_Val_2_H^+^, *m/z* 865. Columns are arranged from (bottom) lowest to (top) highest [Val]; horizontally by chiral composition, with (far left) L-Ser/L-Val and (far right) D-Ser/D-Val. In the mismatched chirality cases, a third substitution can be observed at low abundance. It is notable that this is not observed in the cases where Val and Ser chirality match.

Figure S28: Array of representative mass spectra showing Phe substitution

Phe gave the substituted octamers, Ser_7_PheH^+^, *m/z* 901, and Ser_6_Phe_2_H^+^, *m/z* 961. Columns are arranged from (bottom) lowest to (top) highest [Phe]; horizontally by chiral composition, with (far left) L-Ser/L-Phe and (far right) D-Ser/D-Phe. The peak *m/z* 908.5 is assigned to [11Phe+2H]^2+^, see tandem mass spectrum in **Figure S29**.

Figure S29: MS/MS of Phe-related cluster *m/z* 908.5

Product ion mass spectrum confirming assignment of *m/z* 908.5 (see **Figure S13**) as [11Phe+2H]^2+^. The cluster is very fragile and only a fraction survives the isolation step, giving low signal 1.8E1 counts.

Figure S30: Scatter plot of isoLeu substitution

Scatter plots showing the relative abundance of Ser_8_ and isoLeu-substituted octamers with varying [isoLeu]. When Ser and Leu chirality match (**a-b**), substitution is more favored at lower [isoLeu] compared to the mismatched chirality (**e-f**) and the intermediate effect obtained with racemic isoLeu (**c-d**). As in the case of Leu (main text **Fig. 3**), double substitution is comparatively more abundant than single substitution with mismatched Ser and isoLeu (**e-f**).

Figure S31: Scatter plot of Ala substitution

Scatter plots showing the relative abundance of Ser_8_ and Ala-substituted octamers as [Ala] is varied.

Figure S32: Scatter plot of norVal substitution

Scatter plots showing the relative abundance of Ser_8_ and norVal-substituted octamers as [norVal] is varied. When Ser and Leu chirality match (**a-b**), Ser_7_norValH^+^ is more abundant than the Ser_6_norVal_2_H^+^, when the chirality of Ser and Leu do not match (**e-f**), the double substitution becomes more abundant, as in the case of Leu and Val (main text **Figs. 3 & 4**).

References

1.	Cooks RG, Zhang D, Koch KJ, Gozzo FC, Eberlin MN. Chiroselective Self-Directed Octamerization of Serine:  Implications for Homochirogenesis. Analytical Chemistry. 2001;73(15):3646-55. doi: 10.1021/ac010284l.

2.	Julian RR, Hodyss R, Kinnear B, Jarrold MF, Beauchamp JL. Nanocrystalline Aggregation of Serine Detected by Electrospray Ionization Mass Spectrometry:  Origin of the Stable Homochiral Gas-Phase Serine Octamer. The Journal of Physical Chemistry B. 2002;106(6):1219-28. doi: 10.1021/jp012265l.

3.	Takats Z, Nanita SC, Cooks RG, Schlosser G, Vekey K. Amino Acid Clusters Formed by Sonic Spray Ionization. Analytical Chemistry. 2003;75(6):1514-23. doi: 10.1021/ac0260793.

4.	Nemes P, Schlosser G, Vékey K. Amino acid cluster formation studied by electrospray ionization mass spectrometry. Journal of Mass Spectrometry. 2005;40(1):43-9. doi: <https://doi.org/10.1002/jms.771>.

5.	Nanita SC, Cooks RG. Negatively-Charged Halide Adducts of Homochiral Serine Octamers. The Journal of Physical Chemistry B. 2005;109(10):4748-53. doi: 10.1021/jp046653+.

6.	Jordan JS, Williams ER. Dissociation of large gaseous serine clusters produces abundant protonated serine octamer. Analyst. 2021;146(8):2617-25. doi: 10.1039/D1AN00273B.

7.	Shira BA, Cooks RG. Serine Octamer Clusters Direct the Chirality of Peptides Formed in Water Microdroplets. The Journal of Physical Chemistry A. 2025;129(20):4535-42. doi: 10.1021/acs.jpca.5c00473.

8.	Unni K, Shira BA, Holden DT, Pradeep T, Cooks RG. Serine Octamer Substitution Reactions With α-Hydroxy Acids. Rapid Communications in Mass Spectrometry. 2026;40(18):e70133. doi: <https://doi.org/10.1002/rcm.70133>.

9.	Kelly MA, Vestling MM, Fenselau CC, Smith PB. Electrospray analysis of proteins: A comparison of positive-ion and negative-ion mass spectra at high and low pH. Organic Mass Spectrometry. 1992;27(10):1143-7. doi: <https://doi.org/10.1002/oms.1210271028>.

10.	Kharlamova A, Prentice BM, Huang T-Y, McLuckey SA. Electrospray Droplet Exposure to Gaseous Acids for the Manipulation of Protein Charge State Distributions. Analytical Chemistry. 2010;82(17):7422-9. doi: 10.1021/ac101578q.
